# The synergistic effects of *TaAGP*.*L-B1* and *TaSSIVb-D* mutations in wheat lead to alterations of gene expression patterns and starch content in grain development

**DOI:** 10.1371/journal.pone.0223783

**Published:** 2019-10-11

**Authors:** Shunlin Zhang, Huijun Guo, Ahsan Irshad, Yongdun Xie, Linshu Zhao, Hongchun Xiong, Jiayu Gu, Shirong Zhao, Yuping Ding, Luxiang Liu

**Affiliations:** Institute of Crop Sciences, Chinese Academy of Agricultural Sciences, National Engineering Laboratory of Crop Molecular Breeding, National Center of Space Mutagenesis for Crop Improvement, Beijing, China; Murdoch University, AUSTRALIA

## Abstract

Starch is synthesized from a series of reactions catalyzed by enzymes. ADP-glucose pyrophosphorylase (AGPase) initiates the synthesis pathway and synthesizes ADP-glucose, the substrate of starch synthase (SS), of which SSIV is an isoform. Mutations of the AGPase subunit and SSIV-coding genes affect starch content and cause variation in the number of granules. Here, we pyramided the functional mutation alleles of the AGPase subunit gene *TaAGP*.*L-B1* and the SSIV-coding gene *TaSSIVb-D* to elucidate their synergistic effects on other key starch biosynthesis genes and their impact on starch content. Both the *TaAGP*.*L-B1* and *TaSSIVb-D* genes were expressed in wheat grain development, and the expression level of *TaAGP*.*L-B1* was higher than that of *TaSSIVb-D*. The *TaAGP*.*L-B1* gene was downregulated in the *agp*.*L-B1* single and *agp*.*L-B1/ssIV-D* double mutants at 12 to 18 days after flowering (DAF). *TaSSIVb-D* expression was significantly reduced at 6 DAF in both *ssIV-D* single and double mutants. In the *agp*.*L-B1/ssIV-D* double mutant, *TaGBSSII* was upregulated, while *TaAGPSS*, *TaSSI*, and *TaSBEII* were downregulated. Under the interaction of these genes, the total starch and amylopectin contents were significantly decreased in *agp*.*L-B1* and *agp*.*L-B1/ssIV-D* mutants. The results suggested that the mutations of *TaAGP*.*L-B1* and *TaSSIVb-D* genes resulted in variation in the expression patterns of the other four starch synthetic genes and led to a reduction in starch and amylopectin contents. These mutants could be used further as germplasm for resistant starch analysis.

## Introduction

Starch, which consists of amylose and amylopectin, provides carbohydrates for plant and animal growth as well as human life. Both amylose and amylopectin are polymers that are composed of glucose residues by α-1,4-glycosidic bonds and branched by α-1,6-glycosidic bonds, while amylopectin consists of many more branches than does amylose [1]. Amylopectin is the major component of storage starch in crop grains, and the ratio of amylose and amylopectin affects the processing quality of grains. Higher amylose content results in higher resistant starch content [2], and resistant starch can be added to foods to increase dietary fiber because of the very slow rate at which it is digested.

In plants, starch biosynthesis is catalyzed by a series of enzymes, including ADP-glucose pyrophosphorylase (AGPase), starch synthase (SS), starch branching enzyme (SBE) and starch debranching enzyme (DBE). AGPase is thought to be the initiation enzyme and plays important roles in both source and sink starch metabolism [3]. In wheat, AGPase consists of two large subunits (AGP-L) and two small subunits (AGP-S) [4]. The maize AGP-L-coding gene enhances both photosynthetic rates and plant productivity, with the plant biomass and grain yield of transgenic wheat lines increasing by more than 30% [5] and photosynthetic rates significantly increasing in the early seed development stage under high light conditions [6]. Overexpression of the wheat AGP-L gene has improved AGPase activity by more than 1.5-fold, and grain weight has been observed to increase in some transgenic plant lines [7]. Variations in the sequence of the AGP-L gene in the B subgenome (*TaAGP*.*L-B1*) are more abundant than those in the other two subgenomes [8, 9], and the SNPs of *TaAGP*.*L-B1* in the natural population result in variation in thousand grain weight and seed number, but no significant variation is observed in total starch content [9]; however, the mutant line E3-1-3 carrying the missense mutation of *TaAGP*.*L-B1* shows a marked decline in total starch content [10].

Four soluble SSs, SSI, SSII, SSIII and SSIV, are involved in and responsible for amylopectin synthesis in plants; among them, SSIV is believed to use ADP-glucose as the substrate to catalyze the biosynthesis of starch granules [11, 12], and the sequences of the SSIV-coding gene in Arabidopsis, rice and wheat are homologous and have the conserved ADP-glucose binding site [13]. The *SSIV* gene is involved in the starch granule initiation process and controls starch granule number and shape in plants. In the *ssIV* mutant of Arabidopsis, decreased starch granule number leads to an increase in AGPase [14] and ADP-glucose accumulation [12], and wheat *ssIV* mutants also exhibit a decrease in the number of starch granules in the chloroplasts, affecting photosynthesis [15]. Additionally, the granules of rice *ssIV* mutants are changed from a polyhedral to a spherical shape [16]. Moreover, enhanced *AtSSIV* expression and SSIV activity in potatoes lead to a significant increase not only in transitory granules but also in storage starch content, producing pleiotropic effects on the upregulated AGPase with higher ADP-glucose content [17], which implies that variation in SSIV activity would affect AGPase and that these enzymes might interact with the starch biosynthesis pathway.

Through EMS treatment and TILLING selection, functional mutation lines are identified for the key starch biosynthesis genes *TaAGP*.*L-B1* and *TaSSIVb-D*, which are homologous genes of *TaAGP*.*L* and *TaSSIVb* in the B and D subgenomes, respectively. The functional mutant line E3-1-3, which carries a missense mutation of *TaAGP*.*L-B1* gene, results in a significant decrease on the expression level of *TaAGP*.*L-B1* gene in developmental seeds and a significant decline on total starch content [10]. The functional mutant line of *TaSSIVb-D* gene carries a missense mutation, which causes a significant decrease on the starch granule number in chloroplasts of leaves [15], both mutant alleles are pyramided by crossing. Using the pyramided lines as materials, *TaAGP*.*L-B1*, *TaSSIVb-D* and other key starch biosynthesis gene expression patterns were analyzed in the current paper, and their interaction during wheat grain development and effects on starch and amylopectin contents were studied. The results deepen our knowledge of *TaAGP*.*L-B1* and *TaSSIVb-D* genes in the starch biosynthesis pathway.

## Materials and methods

### Materials

The seeds of winter wheat variety Jing 411 were treated with EMS chemical mutagen, and the M_2_ population was developed by the single-seed descent method. Point mutations on target genes *TaAGP*.*L-B1* and *TaSSIVb-D* were further screened in M_2_, and mutant lines E3-1-3 and E1137, which carry the functional mutation alleles *agp*.*L-B1* and *ssIV-D*, respectively, were identified through TILLING [10, 15]. Then, E3-1-3 and E1137 were crossed to develop the F_2_ population, and the F_2_ individuals carrying no (labeled as wild type, WT), single (*agp*.*L-B1* or *ssIV-D*) or double (*agp*.*L-B1*/*ssIV-D*) mutation alleles were further used for gene expression analysis. Each individual was further advanced into F_3_, and their grains were used to determine the total starch and amylose contents.

All lines were planted in the field at the experimental station of the Institute of Crop Sciences, Chinese Academy of Agricultural Sciences, during the normal growing season with normal management. The day that the first spikelet started flowering was marked as the flowering day of the spike, and the grains were sampled at 6-day intervals from 6 days after flowering (DAF) until 30 DAF. Each sample was collected at 4 p.m. and included 2–3 grains from the spikelet in the middle of the spike, three biological replications were performed for each genotype and samples were stored at -80°C for RNA extraction. Matured grains of each genotype were harvested for starch content measurement with 2 biological replications and 2 technical replications.

### Genotyping identification

Genotyping of each line was identified through Kompetitive allele-specific PCR (KASP) or sequencing. Lines carrying mutant allele of *TaAGP*.*L-B1* gene were identified through KASP, the specific forward KASP primer sequence was CCCAAAGTTCGAGTTTTATGATC[T/C], and the reverse one was AATAGTCTGAATGGCAGGTTGA. PCR reaction and running protocols of KASP was followed by standard KASP guidelines at website http://www.lgcgenomics.com. Lines carrying mutant alleles of *TaSSIVb-D* gene were detected directly by sequencing after PCR amplification with a set of genome-specific primer [15].

### RNA extraction

RNA from each sample was extracted with a Plant Total RNA Purification Kit (GeneMark, TR02, Taiwan) according to the manufacturer’s instructions, and the concentrations were measured with a NanoDrop 2000 spectrophotometer. Each genotype included three biological replications.

### Quantitative real-time PCR (qRT-PCR)

A total of 1 μg of RNA was used to synthesize the cDNA with a TransScript Kit (TransGen, AT341) according to the manufacturer’s instructions, and the concentration of cDNA was quantified with a NanoDrop 2000 spectrophotometer and made uniform for qRT-PCR.

qRT-PCR was performed with the TransStart SuperMix Kit (TransGen, AQ131) and ran on a CFX96 system (Bio-Rad Co., USA). The amplification process was initiated at 94°C for 30 s, followed by 43 cycles of denaturing for 5 s, annealing for 15 s, and extension for 10 s and then a melting curve stage. Three biological replications with three technical replications were performed for each genotype, and *Actin* (GenBank accession no: AAW78915), a housekeeping gene, was used as an internal control. The ΔΔ*Ct* method was used to analyze the relative expression level, the expression level of *TaSSII* gene at 6 DAF in WT was used as a control with the value of 1, those of other genes in each sample were compared with it, and the relative expression value was calculated by Microsoft Excel.

The expression patterns of genes involved in starch biosynthesis were analyzed, including *TaAGP*.*L-B1*, *TaSSIVb-D*, *TaAGPSS*, *TaSSI*, *TaSSII*, *TaSSIII*, *TaSBEII*, *TaISA-1*, *TaGBSSI* and *TaGBSSII*. Primers for qRT-PCR are listed in S1 Table.

### Starch content measurement

Mature grains were milled by a cyclone mill (FOSS Analytical, Co., Ltd, CT410) with a 0.5 mm screen. The grain total starch and amylose contents of each genotype were measured with two biological and two technical replications according to National Standards NY/T11-1985 and NY/T55-1987 at the Cereal Quality Supervision and Testing Center, Ministry of Agriculture, China, and the percentage of amylopectin was calculated as 100% minus the percentage of amylose.

### Data analysis

Statistical analysis was conducted by one-way ANOVA, and *P* < 0.05 or 0.01 was considered significant based on Student’s *t*-test.

## Results

### Expression patterns of *TaAGP*.*L-B1* and *TaSSIVb-D* in wheat grain development of *agp*.*L-B1/ssIV-D* single and double mutants

**Fig 1:**
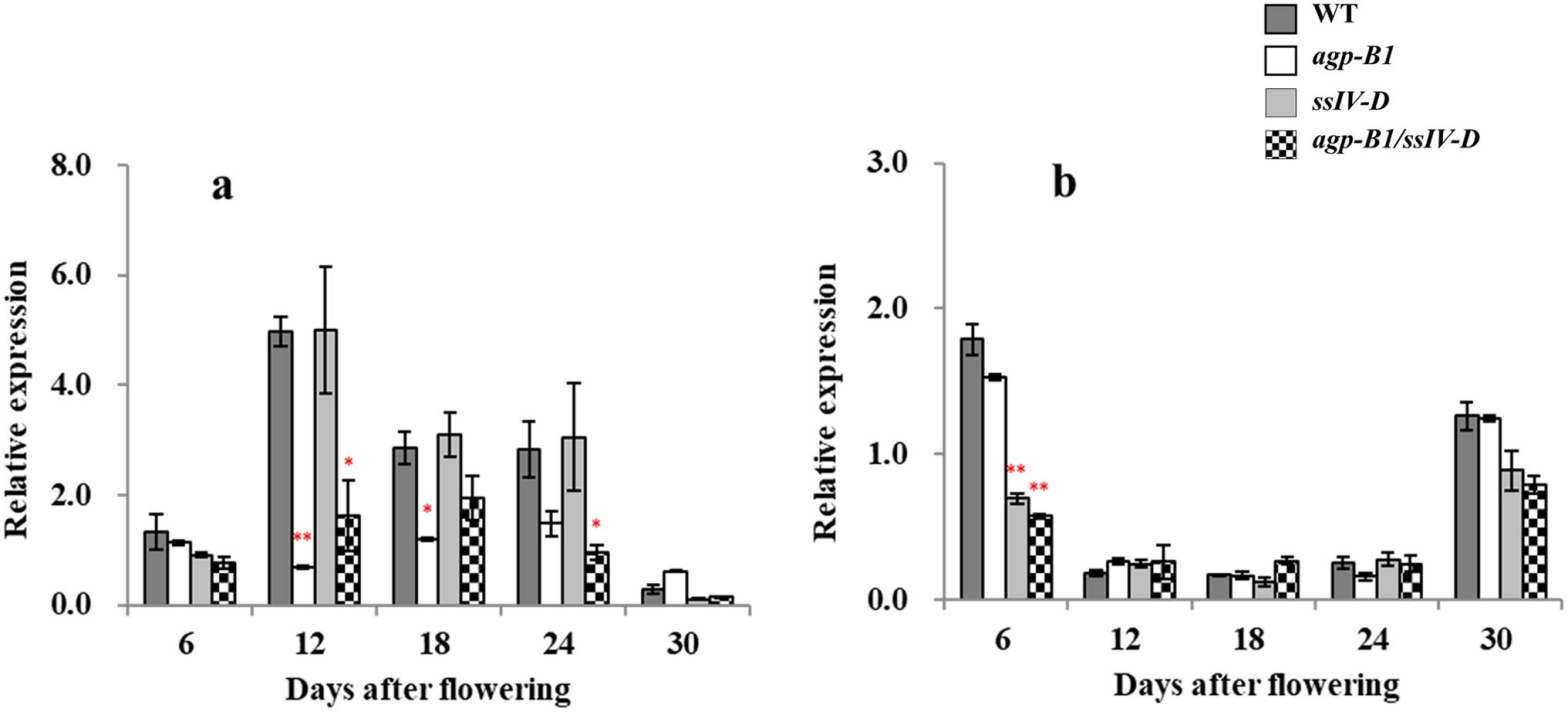
Expression pattern of the *TaAGP*.*L-B1* and *TaSSIVb-D* genes in developing wheat grains of *agp*.*L-B1/ssIV-D* single and double mutants. a, relative expression level of *TaAGP*.*L-B1* gene; b, relative expression level of *TaSSIVb-D*. * and ** mean significantly different from WT at the *P* = 0.05 or 0.01 level respectively, based on Student’s *t*-test.

In wheat grain development, the expression pattern of both genes, *TaAGP*.*L-B1* and *TaSSIVb-D*, varied at the five sampling stages, which started from 6 DAF, and the expression levels of *TaAGP*.*L-B1* were relatively higher than those of *TaSSIVb-D* (Fig 1). *TaAGP*.*L-B1* was highly expressed from 12 to 24 DAF and significantly downregulated (1/8~1/2-fold) in the *agp*.*L-B1* single and double mutants compared to in the nonmutants, the WT and the *ssIV-D* single mutant (Fig 1a), while there was no significant difference between *agp*.*L-B1* single and double mutants The *TaSSIVb-D* expression level was relatively higher at 6 and 30 DAF than at the other three stages and showed a significant reduction (approximately 1/3-fold) at 6 DAF in both *ssIV-D* single and double mutants (Fig 1b), and the expression also showed no significant differences between *ssIV-D* single and double mutants.

### *TaAGP*.*L-B1* and *TaSSIVb-D* mutations affected the expression patterns of other key starch biosynthesis genes in wheat grain development

**Fig 2:**
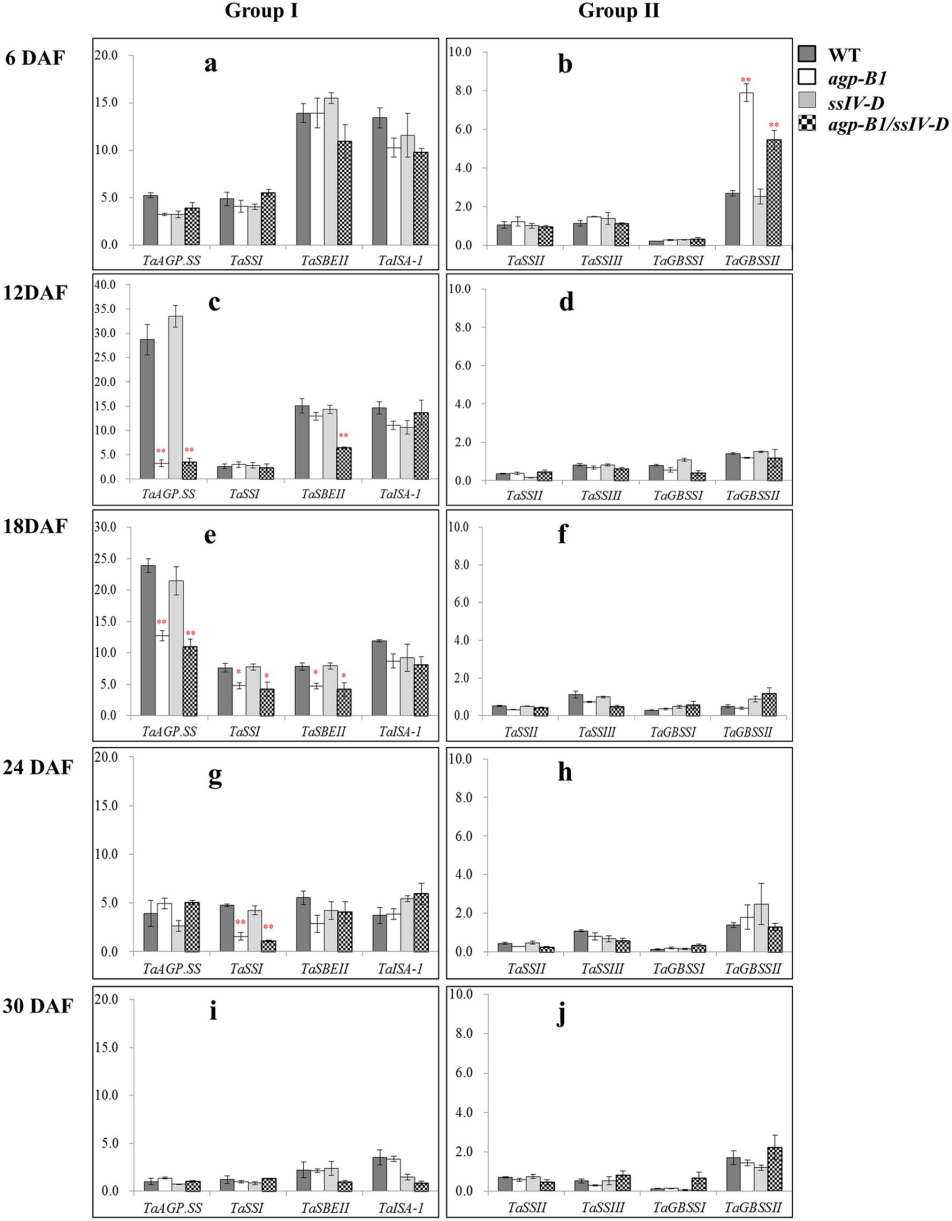
Expression pattern of key starch biosynthesis genes in developing wheat grains of *agp*.*L-B1/ssIV-D* single and double mutants. The genes were categorized into two groups based on relative expression levels. Genes in group I (a, c, e, g, i) showed higher expression level than those in group II (b, d, f, h, j). *, ** indicate significantly different from WT at 0.05 or 0.01 level respectively, based on Student’s *t*-test.

According to their expression levels in grain development, the other eight key genes were categorized into two groups. Group I, which included four genes, *TaAGPSS*, *TaSSI*, *TaSBEII* and *TaISA-1*, exhibited higher expression levels, while Group II showed lower expression levels and contained the other four genes, *TaSSII*, *TaSSIII*, *TaGBSSI* and *TaGBSSII* (Fig 2).

In Group I, all four genes showed higher expression levels at the earlier developmental stages and the lowest levels at 30 DAF compared with the WT. In addition, the expression patterns of *TaAGPSS*, *TaSSI* and *TaSBEII* were altered in the *agp*.*L-B1* mutants at the middle developmental stages, while those of *TaISA-1* were not changed significantly; however, no gene showed significant variation in the *ssIV-D* single mutant at any developmental stage (Fig 2a, 2c, 2e, 2g and 2i). *TaAGPSS* was more highly expressed at 12 and 18 DAF, and its expression was significantly altered between WT and *agp*.*L-B1* single and double mutants at the two stages, especially at 12 DAF, with that in the mutants being were only 1/9~1/2 of that in the WT (Fig 2c and 2e), but there were no significant differences between *agp*.*L-B1* single and double mutants. The expression of the *TaSSI* gene was significantly affected at 18 and 24 DAF as well, being approximately 1/4-1/2 that of the WT (Fig 2e and 2g). *TaSBEII* showed significant reductions by 70.5% and 53.9% in the *agp*.*L-B1/ssIV-D* double mutant only at 12 and 18 DAF, respectively, and no difference was detected at other stages.

In Group II, the expression levels of *TaSSII*, *TaSSIII* and *TaGBSSI* were relatively lower than those of *TaGBSSII* at all investigated stages, but these differences were not significant (Fig 2b, 2d, 2f, 2h and 2j). *TaGBSSII* was upregulated more than 2-fold at 6 DAF in the *agp*.*L-B1* and *agp*.*L-B1/ssIV-D* mutants compared with the WT and was the only upregulated gene in the mutants (Fig 2b).

### Starch and amylopectin content variation in the *agp-B1/ssIV-D* mutants

Starch content in both *agp-B1* and *agp-B1/ssIV-D* mutants showed a significant reduction, and the decrease in the *agp-B1/ssIV-D* double mutant was greater than that in the *agp-B1* single mutant. The amylopectin content in the *agp-B1/ssIV-D* double mutant was significantly decreased compared with that of the WT, and no remarkable changes were observed in the other two single-mutant genotypes (Table 1).

**Table 1:** The grain starch and amylopectin contents in the single and double mutants of *agp-B1/ssIV-D*.

Genotype	Starch Content (%)	Amylopectin Content (%)
WT	63.88±0.58	64.73±1.02
*agp-B1*	59.48±0.79 *	62.18±1.02 *
*ssIV-D*	63.30±0.65	65.08±0.09
*agp-B1/ssIV-D*	55.50±1.44 **	60.25±1.43 *

Data were means ± standard deviation;

* and ** indicate significantly different from WT at the *P* = 0.05 or 0.01 level respectively, based on Student’s *t*-test.

## Discussion

### *TaSSIVb-D* and *TaAGP*.*L-B1* mutations affected the expression patterns of other key starch biosynthesis genes

A large set of enzymes are involved in the starch biosynthesis pathway, they catalyze a series of reactions, and sucrose is synthesized into starch. In grains and other storage organs, this reaction is mainly catalyzed by AGPase, SS, granule-bound starch synthase (GBSS), SBE, and DBE [18], and the expression patterns of these enzymes vary among bread wheat grain developmental stages [19]. Mutations in the GBSSI-, SSII- and SBEII-coding genes affect the gene expression patterns of AGPase, isoamylase (ISA) and other enzymes [20, 21], and alterations in amylose content are impacted by variations in the expression patterns of amylose biosynthesis and other starch metabolism genes [22].

**Fig 3:**
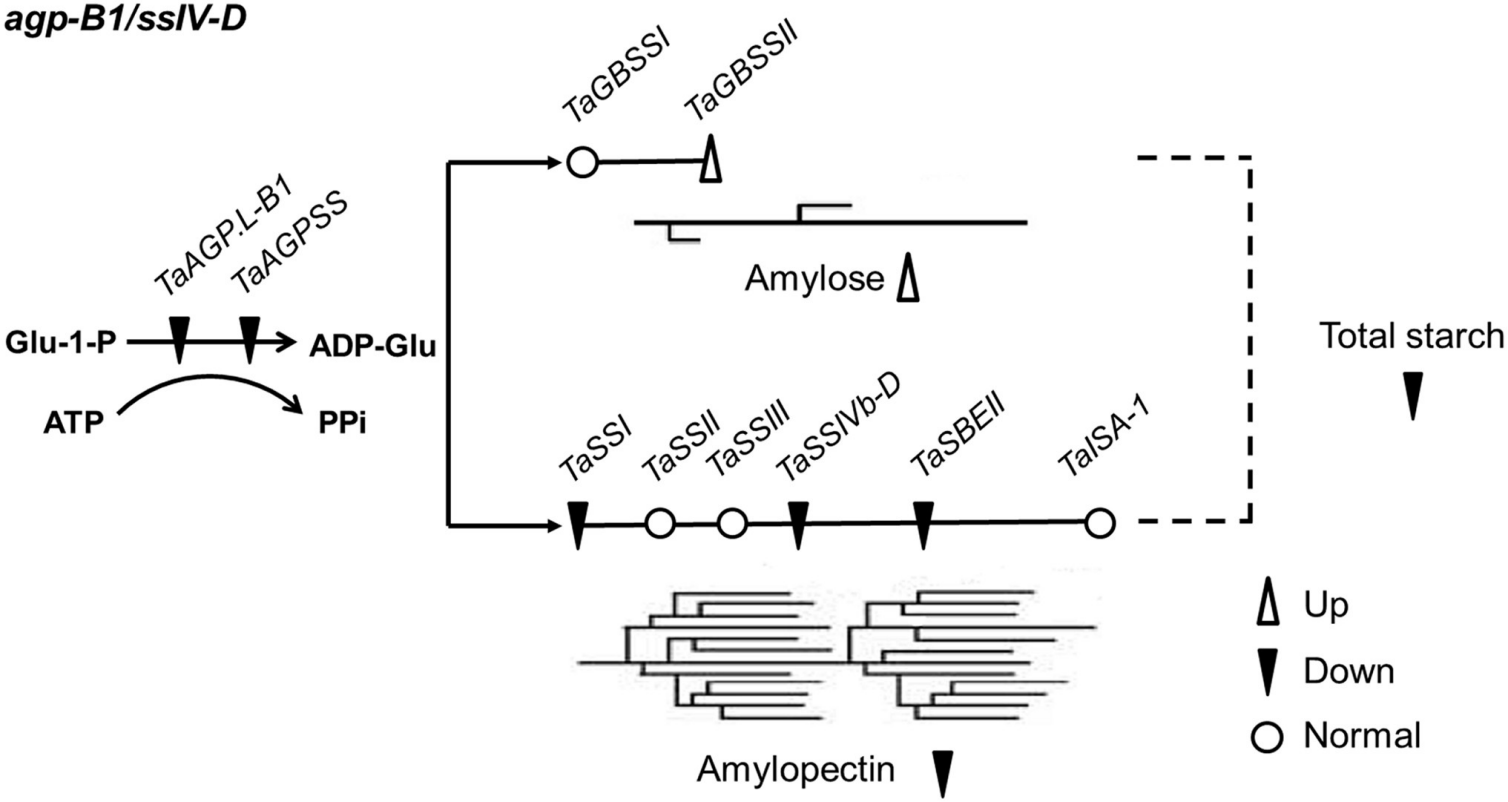
A schematic illustration of the genes involved in starch biosynthesis and their regulation in the *agp-B1/ssIV-D* double mutant.

We identified two mutants of *TaAGP*.*L-B1* and *TaSSIVb-D*, which are the coding genes of AGPase and SSIV, respectively, and contribute to starch granule number and grain starch content [10, 15]. AGPase is the initiation enzyme of starch synthesis [23], and SSIV is one of the isoforms of the SS family [18]. Moreover, SS isoforms interact with SBEs [24], and altered *SSIV* gene expression has pleiotropic effects on AGPase [17]. In our *agp-B1/ssIV-D* double mutant, the expression levels of *TaGBSSII*, *TaAGP*.*SS*, *TaSBEII* and *TaSSI* were significantly altered by *TaSSIVb-D* and *TaAGP*.*L-B1* mutations (Fig 3), and the range of variation in the double mutant was greater than that in the single mutants, which implied that *TaAGP*.*L-B1* and *TaSSIVb-D* had synergistic effects during grain development.

As a hexaploid specie, many genes in wheat have three functionally redundant copies, and mutation effect of one homoeolog might be compensated by the other two. For example, *TaSBEII* gene has three homoeologous in wheat genome, the amylose content did not show any differences between the wild type and the single mutant of *TaSBEII* gene; however, it exhibited a significant increase in the mutants combining all three homoeologous genes [25]. Both *TaAGP*.*L* and *TaSSIVb* genes have three homoeologous in the A, B and D subgenomes respectively, it has been reported that single mutations in *TaSSIVb-D* and *TaAGP*.*L-B1* resulted in significant phenotypic alterations respectively, and without compensatory variation in the other two homoeologous genes [10, 15], which suggest that not only double and triple mutants, but also single mutants could lead to phenotypic alterations in hexaploid wheat.

Genotype is one of the very important factors affecting gene expression [26]. The four genotypes we used in the experiments were from F_2_ individuals, which eliminated background effects, so the expression variations observed resulted from the *TaSSIVb-D* and *TaAGP*.*L-B1* mutations.

### The genes involved in starch biosynthesis in wheat grain development

The coding genes of AGPase, GBSS, SS and SBE control grain starch synthesis (Fig 3), are expressed at the wheat grain development stage, and their expression patterns ranged by genes and genotypes. In grain, the expression levels of small subunit coding gene of AGPase were highest at 15 DAF [27] or highly expressed throughout the whole grain development stages [19], while in pericarp, its peak was detected at 6 DAF, so did *GBSSII* [28]. SSI and ISA genes were highly expressed at early and middle stages [19], expression peaks of different isoforms of SBEs and SSs ranged slightly, but all showed a down-up-down expression pattern [26]. It has reports showed that all the above mentioned genes reached their expression peaks at approximately 12 to 18 DAF [29–31], our results were identical to this trend. The expression levels of *TaGBSSII* were relatively higher than those of *TaGBSSI* in our genotypes, while in other reported genotypes, *TaGBSSI* expression was 60 times higher than that of *TaGBSSII* [29], which might be due to genotypic differences. As one of the DBEs, ISA is expressed in the endosperm and influences amylopectin structure [32], and the *TaISA-1* gene in the present study was relatively more highly expressed, but no difference was detected in the mutants. It has been reported that the expression level of *TaSSIVb* is not affected by grain genotype [20], but in our *ssIV-D* single and double mutants, the homologous gene of *TaSSIVb* in the D subgenome showed significant variance that was the direct impact of the *TaSSIVb-D* gene mutation. High expression occurred at the initial (6 DAF) and final (30 DAF) stages of grain development, and expression levels at the other three investigated stages were quite low; however, the results showed that *TaSSIVb* was highly expressed throughout the developmental stages of grain [19].

### Synergistic effects of *TaAGP*.*L-B1* and *TaAGP*.*SS* gene expression in wheat grain development

AGPase is known as the rate-limiting catalyzing enzyme in starch biosynthesis, and its complex is composed of the interaction of small and large subunits, which are coded by *TaAGPSS* and *TaAGP*.*L* in wheat [33]. Variation in any subunit could affect AGPase activity and lead to variation in the expression of the other subunit-coding genes. The expression patterns of *TaAGPSS* and *TaAGP*.*L-B1* were similar in the present study; the expression of both was higher at the middle stage and lower at the initial and final stages of grain development and showed significant reduction at the stage of highest expression in *agp*.*L-B1* single and *agp-B1/ssIV-D* double mutants. Expression variation in AGPase-coding genes impacts the starch content in wheat [6, 7, 10, 27]. Overexpression of large or small subunit genes remarkably enhances the activity of AGPase as well as starch biosynthesis in the endosperm of wheat [7, 27], and downregulation of large subunit-coding genes results in a significant decrease in the starch content of wheat grains [10]. Both *TaAGPSS* and *TaAGP*.*L-B1* were downregulated by up to 9-fold during the highest expression periods at 12 to 18 DAF in *agp*.*L-B1* single and double mutants, which might be the main reason for the starch content decrease in both mutants.

### The amylopectin content was significantly reduced in the *agp-B1/ssIV-D* double mutant

Amylopectin is mainly synthesized before 12 DAF in wheat grain under the catalyzing reactions of SS, SBE, and DBE enzymes [18, 34], while amylose is catalyzed by GBSS. The amylopectin contents were significantly reduced in SBE, SSI and GBSS mutants [35–39]. Reduced transcription of *TaSBEII* resulted in up to a 2-fold increase in amylose [40], which indicated a remarkable reduction in amylopectin. The *TaSBEII* was downregulated at 12 and 18 DAF in the *agp-B1/ssIV-D* double mutant. Decreased amylopectin content and chain length were detected in the *TaSSI* suppression mutants of rice and wheat [37–39], and *TaSSI* was differentially expressed in our double mutants at 18 and 24 DAF. Both genes *TaSBEII*, and *TaSSI* may contribute to the decreased amylopectin content in *agp-B1/ssIV-D* double mutants and indirectly lead to increased amylose content, which is positively related to resistant starch content. While the *TaGBSSII* gene responsible for amylose synthesis was highly expressed at 6 DAF and significantly upregulated in *agp-B1* and *agp-B1/ssIV-D* mutants, and may contribute negatively to the decreased amylopectin content.

## Supporting information

S1 Table The primer sequences of each gene used for qRT-PCR amplification. (PDF)
