# Supplementary material for: The synergistic effects of TaAGP.L-B1 and TaSSIVb-D mutations in wheat lead to alterations of gene expression patterns and starch content in grain development
Source: PLoS One. 2019 Oct 11;14(10):e0223783. doi: 10.1371/journal.pone.0223783 (PMC6788705; doi:10.1371/journal.pone.0223783)
Supplement: S1 Table — (PDF) [file pone.0223783.s001.pdf]

S1 Table. The primer sequences of each gene for qRT-PCR amplification

Gene	Primer sequence		Reference
	Forward primer	Reverse primer	
TaSSIVb-D	CCGCTTCCAAGCATGTAACAGC	CCAGTAACCATCCATCAATTAG	[15]
TaAGP.L-B1	GAACTACATGACTTTGGGTCTGAGA	CATCATCATCGCATTCTTGAGCT	[10]
TaAGPSS	GCAAGATACACCATTCAGTAGTTGGAC	GACTGTTCCACTAGGGAGTAAAGCATC	[19]
TaSSI	GTTTCCCACAGAATAACTGCAGGTTGC	GTATGGTCTTTCGTCATGCCTCGC	[19]
TaSSII	GAATCAAGTTGGCGAGGATGTCGAC	CAATCTCTTTGCCCTCTGAAACTGCAT	[19]
TaSSIII	GACTTCTCAGGAAATGTCTCTAGCAG	ACCAGTGCACTTATTCTGACACGGTAC	[19]
TaSBEII	GCACCAGTATGTTTCACGGAAACATG	GGCATACACGACCGCAGTTCTG	[19]
TaISA-1	GTCTTCAACCATACAGCTGAGGGT	GAACCTCTGGTCATTATGGATGCAAG	[19]
TaGBSSI	CTCGCCGCCAACTACGACGTC	TGCTCGGGAACTTCTCCTCCAC	[19]
TaGBSSII	GTTCCAAGTAGGTTCGAGCCATGT	AGAGCCTCCTCCCACTTCTTTGC	[19]
